# Characteristics of COVID-19 Cases and Outbreaks at Child Care Facilities — District of Columbia, July–December 2020

**DOI:** 10.15585/mmwr.mm7020a3

**Published:** 2021-05-21

**Authors:** Christine Kim, Sasha McGee, Shreya Khuntia, Azam Elnour, Fern Johnson-Clarke, Anil Mangla, Preetha Iyengar, LaQuandra Nesbitt

**Affiliations:** ^1^Epidemic Intelligence Service, CDC; ^2^Center for Policy, Planning, and Evaluation, Department of Health, Washington, DC; ^3^Department of Health, Washington, DC.

The occurrence of cases of COVID-19 reported by child care facilities among children, teachers, and staff members is correlated with the level of community spread (*1*,*2*). To describe characteristics of COVID-19 cases at child care facilities and facility adherence to guidance and recommendations, the District of Columbia (DC) Department of Health (DC Health) and CDC reviewed COVID-19 case reports associated with child care facilities submitted to DC Health and publicly available data from the DC Office of the State Superintendent of Education (OSSE) during July 1–December 31, 2020. Among 469 licensed child care facilities, 112 (23.9%) submitted 269 reports documenting 316 laboratory-confirmed cases and three additional cases identified through DC Health’s contact tracers. Outbreaks associated with child care facilities,^†^ defined as two or more laboratory-confirmed and epidemiologically linked cases at a facility within a 14-day period (*3*), occurred in 27 (5.8%) facilities and accounted for nearly one half (156; 48.9%) of total cases. Among the 319 total cases, 180 (56.4%) were among teachers or staff members. The majority (56.4%) of facilities reported cases to DC Health on the same day that they were notified of a positive test result for SARS-CoV-2, the virus that causes COVID-19, by staff members or parents.^§^ Facilities were at increased risk for an outbreak if they had been operating for <3 years, if symptomatic persons sought testing ≥3 days after symptom onset, or if persons with asymptomatic COVID-19 were at the facility. The number of outbreaks associated with child care facilities was limited. Continued implementation and maintenance of multiple prevention strategies, including vaccination, masking, physical distancing, cohorting, screening, and reporting, are important to reduce transmission of SARS-CoV-2 in child care facilities and to facilitate a timely public health response to prevent outbreaks.^¶^

During May 29–June 21, 2020, DC Health instituted phase 1 reopening guidance for child care facilities, which recommended daily health screening; mandatory use of cloth or disposable face masks for adults and recommended use for children aged ≥2 years; physical distancing (≥6 ft), especially during meals and naps; limiting the size of classes or cohorts** to ≤10 persons; limiting interactions between cohorts; using partitions between groups; and increasing the frequency of hand hygiene, cleaning, and disinfection of high-touch surfaces. It was recommended that mouthed or soiled toys should be set aside, cleaned, and sanitized before reuse. Proper operation of ventilation systems according to the manufacturer and increased circulation of outdoor air (open windows) were also recommended. On June 22, DC Health instituted phase 2 guidance, in which previous recommendations were changed to requirements and updated to include limiting cohort sizes to 12 persons,^††^ minimizing the use of floating teachers or staff members between classes,^§§^ staggering arrival and departure times for children, requiring masks for children aged ≥2 years (other than those with developmental exceptions), and detailed requirements for reporting cases to DC Health (*4*).

Child care facilities were required to report COVID-19 cases among attending employees, children, or visitors through an online consult form on a dedicated website (*5*). Upon receipt of a report, DC Health investigated the case, shared public health guidance, and identified a list of close contacts^¶¶^ who needed to quarantine (*6*). This study reviewed data, including qualitative notes, from case investigations of child care facilities that reported to DC Health during July 1–December 31, 2020. The analysis also used publicly available data from OSSE*** on child care facilities licensed in DC as of December 31, 2020. During July 1–December 31, a total of 354 reports were submitted by 145 child care facilities (Supplementary Figure, https://stacks.cdc.gov/view/cdc/105818). Most (291; 72%) cases were reported to DC Health on the same day that staff members or parents notified the facility of a positive SARS-CoV-2 test result.^†††^ Of the 354 reports received, 85 were excluded from the analysis for the following reasons: 1) duplicate submissions (14); 2) incomplete investigations because of facility nonresponse or lack of information (17); and 3) incorrect reporting of cases (e.g., household member not attending the facility received a positive SARS-CoV-2 test result [54]). The final analysis included 112 facilities that submitted 269 reports documenting laboratory-confirmed COVID-19 in 316 cases; three additional cases were identified through DC Health’s contact tracing data. Symptomatic persons without laboratory confirmation of infection were not included. This activity was reviewed by CDC and was conducted consistent with applicable federal law and CDC policy.^§§§^

Characteristics of child care facilities, cases, and facility-associated outbreaks were included in the analysis. Modified Poisson generalized linear models with robust error variance were used to estimate the crude risk ratios (RRs) and 95% confidence intervals of facility characteristics associated with outbreak status. P-values ≤0.05 were considered statistically significant. Statistical analyses were conducted using Stata (version 16; StataCorp).

During July 1–December 31, COVID-19 cases were reported from 112 facilities, including 102 (91.1%) center-based facilities and 10 (8.9%) home-based facilities (Table 1). Among facilities with reported cases, 55 (49.1%) had one COVID-19 case, and 30 (26.8%) had two or more cases not identified as outbreak-associated (i.e., not epidemiologically linked within a 14-day period). Twenty-nine index cases from 27 facilities resulted in 127 additional cases that met the outbreak-associated case definition (median of three outbreak-associated cases per index case); 69 (44.2%) outbreak-associated cases were from five facilities with ≥10 cases each. Among 319 total cases reported, 148 (46.4%) were among teachers, 139 (43.6%) were among children, and 32 (10.0%) were among staff members. Sixty-eight (21.3%) persons with COVID-19 were asymptomatic,^¶¶¶^ 43 (63.2%) of whom were children. A total of 1,830 close contacts were identified, with a median of five close contacts per case or 11 per facility (a median of nine close contacts per facility without an outbreak and a median of 27 close contacts per facility with an outbreak). Three facility characteristics were associated with increased risk for an outbreak. First, being in operation for ≤3 years (compared with ≥10 years) was associated with a RR of 3.29. Second, facilities with COVID-19 cases among symptomatic persons who sought testing ≥3 days after symptom onset were at increased risk compared with those in which symptomatic persons sought testing 1–2 days after symptom onset (RR = 2.03). Finally, facilities with asymptomatic cases were at increased risk compared with those without asymptomatic cases (RR = 2.10). Nearly three quarters of overall cases (231; 72.4%) and facility-associated outbreak cases (111; 71.2%) were reported after October 27, 2020, when percentages of positive test results in the community began to increase (Figure).

**TABLE 1:** Characteristics of child care facilities and reported COVID-19 cases* — District of Columbia, July–December 2020

Characteristic	Total (N = 112)^†^	No. (%)		RR (95% CI)^¶^
		Facilities with cases not associated with outbreaks (n = 85)	Facilities with outbreak-associated cases^§^ (n = 27)	
**Total facilities**				
Center-based	102	78 (76.5)	24 (23.5)	Ref
Home-based	10	7 (70.0)	3 (30.0)	1.28 (0.46–3.50)
**No. of years of operation**				
≤3	28	16 (57.1)	12 (42.9)	3.29** (1.38–7.80)
4–9	35	26 (74.3)	9 (25.7)	1.97 (0.77–5.04)
≥10	46	40 (87.0)	6 (13.0)	Ref
**No. of children enrolled**				
≤20	12	10 (83.3)	2 (16.7)	0.59 (0.15–2.28)
21–80	47	35 (74.5)	12 (25.5)	0.90 (0.46–1.77)
>80	46	33 (71.7)	13 (28.3)	Ref
**Average no. of days from symptom onset to SARS-CoV-2 testing**				
0 (same day)	5	5 (100.0)	0	—**^††^**
1–2	58	47 (81.0)	11 (19.0)	Ref
≥3	39	24 (61.5)	15 (38.5)	2.03** (1.04–3.95)
Asymptomatic or presymptomatic during testing	10	N/A	N/A	N/A
**Average no. of days from specimen collection to facility notification of result**				
0 (same day)	11	10 (90.9)	1 (9.1)	Ref
1–2	64	50 (78.1)	14 (21.9)	2.41 (0.35–16.64)
≥3	37	25 (67.6)	12 (32.4)	3.57 (0.52–24.69)
**Average no. of days for facility to report case to DC Department of Health**				
0 (same day)	62	51 (82.3)	11 (17.7)	Ref
1	24	15 (62.5)	9 (37.5)	2.11** (1.00–4.46)
≥2	24	18 (75.0)	6 (25.0)	1.41 (0.58–3.40)
**Asymptomatic cases**				
No	74	61 (82.4)	13 (17.6)	Ref
Yes	38	24 (63.2)	14 (36.8)	2.10** (1.10–4.01)

**Abbreviations:** CI = confidence interval; DC = District of Columbia; N/A = not applicable; Ref = referent; RR = risk ratio.

* A COVID-19 case was defined as a positive nucleic acid amplification test, including positive reverse transcription–polymerase chain reaction test, or positive rapid antigen test result for SARS-CoV-2 in a person who was physically present at a child care facility.

**^†^** Some characteristics might not sum to 112 because of missing values.

^§^ A facility-associated outbreak was defined as two or more laboratory-confirmed COVID-19 cases among children or staff members with onset of illness within a 14-day period, who are epidemiologically linked, do not share a household, and are not listed as a close contact of one another in another setting during standard case investigation or contact tracing.

^¶^ Calculated using a modified Poisson generalized linear model with robust error variance.

** p-value ≤0.05.

**^††^** Category omitted from model because of perfect prediction.

**FIGURE:**
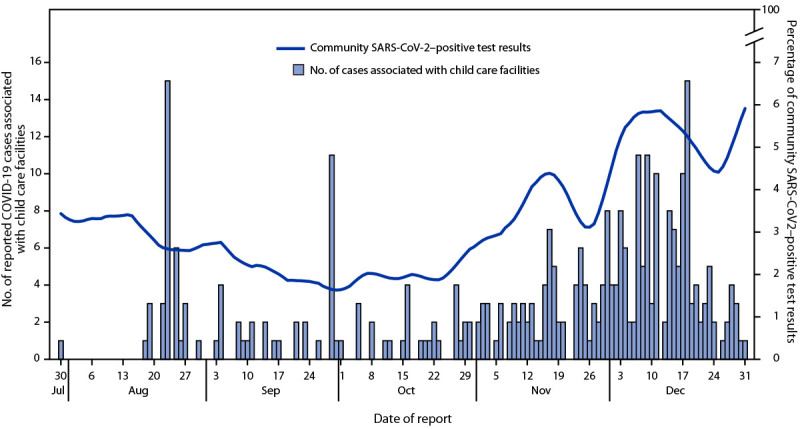
COVID-19 cases associated with child care facilities (N = 319), by date of case report and 7-day moving average percentage of community SARS-CoV-2–positive test results — District of Columbia, July 30–December 31, 2020

The most commonly reported prevention measures implemented by facilities with outbreaks were requirements that masks be worn by teachers and staff members (100%), sending symptomatic employees home immediately (96.3%), limiting class sizes to ≤10 persons (92.6%), and increasing frequency of cleaning and disinfection (74.1%) (Table 2). Facilities with outbreaks often reported difficulty adhering to guidance on symptom monitoring, cohorting, staggered arrival and departure times, limiting physical distance among teachers or staff members (e.g., congregating before classes or carpooling), and minimizing floating of teachers or staff members between classes because of staffing shortages.

**TABLE 2:** Prevention measures, risk factors, and challenges associated with prevention of COVID-19 transmission reported during investigations of child care facilities with COVID-19 outbreaks (n = 27)* — District of Columbia, July–December 2020

Prevention measures, risk factors, and challenges	No. (%)
**Prevention measure**	
Masks worn by teachers and staff members	27 (100.0)
Teachers or staff members with symptoms sent home immediately	26 (96.3)
Cohort size limited to ≤10 persons^ⴕ^	25 (92.6)
Increased daily cleaning and disinfection	20 (74.1)
Temperature monitoring	17 (63.0)
Symptom monitoring of children and staff members	15 (55.6)
No interactions between cohorts	14 (51.9)
Cleaning and disinfecting by third party	10 (37.0)
Staggered arrival and departure times	2 (7.4)
**Risks and challenges**	
Limited physical distancing within cohort	27 (100.0)
Limited physical distancing among teachers or staff members from different cohorts	15 (55.6)
Teachers or staff members floated^§^ between classrooms	10 (37.0)
Break room available for teachers and staff members	4 (14.8)
Siblings in multiple affected cohorts	3 (11.1)
Interactions between separate cohorts	2 (7.4)
External playdates among children of same or different cohorts from child care facility	2 (7.4)
Symptomatic teachers or staff members told to continue working	1 (3.7)

*All guidance recommendations are not included in this table because they might not have been documented during case investigations. Only facilities with an outbreak are included.

^ⴕ^ Cohort size limit increased to 12 persons during phase 2 guidance updated in mid-December 2020 (https://coronavirus.dc.gov/sites/default/files/dc/sites/coronavirus/page_content/attachments/Phase_Two_COVID-19_DC_Health_Guidance_For-Childcare_2021.01.15_FINAL.pdf).

^§^ Floating teachers or staff members are those who leave their assigned cohort to cover for another teacher or staff member who is absent or on break.

## Discussion

This study found limited occurrence of facility-associated outbreaks within DC child care facilities. One quarter of licensed child care facilities reported at least one case; however, facility-associated outbreaks occurred in 27 (5.8%) facilities, accounting for approximately one half of total cases reported from child care facilities (approximately one half of which were reported from five facility-associated outbreaks). Child care facilities in DC were able to adhere to many recommended prevention measures and reporting requirements to prevent the spread of COVID-19.

As has been observed in other studies, the rise in COVID-19 cases and outbreaks among these facilities correlated with the level of community spread (*1*). Although most facilities reported one or two isolated cases during the study period, overall, five close contacts per case were identified in child care facilities, compared with 1.2 per case from a study on community-level contact tracing during a similar period (*7*). Delays of ≥3 days in seeking testing of symptomatic persons was associated with outbreaks. The large number of close contacts identified in these facilities and extended exposure to symptomatic persons might have increased the likelihood of spread and delayed notification and public health response; symptom monitoring for early isolation and diagnosis upon symptom onset is critical to reduce outbreak-associated cases.

Approximately 20% of cases occurred in asymptomatic persons, and most asymptomatic cases were in children, which is similar to findings from a Wisconsin report describing outbreaks in schools (*2*). In addition, outbreaks associated with child care facilities typically involved a large proportion of asymptomatic cases, underscoring the importance of implementing a combination of prevention strategies, including quarantine of close contacts, to prevent outbreaks. Outbreak-associated cases were more likely to occur in facilities operating for ≤3 years, compared with those in facilities operating for ≥10 years. Older, more established facilities might have increased resources or experience in implementing infectious disease prevention measures (*8*). Implementing prevention measures requires resources, and additional revenue losses also might occur because of decreased enrollment and insufficient staffing following quarantining of children, teachers, or staff members (*8*). DC Health’s guidance recommended that teachers and staff members not float between classrooms, but some facilities reported continuing the practice because of challenges associated with staffing shortages. For facilities with subsidized child care services, OSSE introduced a Public Health Emergency Subsidy Rate in January 2021 to offset increased costs or reduced revenues associated with the pandemic (*8*).

The findings in this report are subject to at least five limitations. First, data come from child care facility–based case investigations at a single time point and might miss secondary cases. Although facilities were required to report every case, if subsequent cases were not reported, DC Health was unable to account for residents of other jurisdictions, or those who received tests in other jurisdictions. Second, cases might be underestimated because symptomatic persons who did not have laboratory confirmation of COVID-19 were not included. Third, asymptomatic persons identified through investigations are also likely underestimated because asymptomatic contacts were unlikely to receive tests. Fourth, verifying prevention measures implemented and risks or challenges documented was not possible. Finally, data on whether child care facilities closed and reopened during the study period were not available, and information on prevention measures was more readily available for facilities with outbreaks because of the more in-depth investigations that took place.

Similar to outbreaks reported in school settings (*2*,*9*,*10*), those associated with child care facilities, including outbreak-associated cases, remained low. Implementation and maintenance of multiple prevention strategies, including vaccination, masking, physical distancing, cohorting, screening, and reporting, are important to reduce transmission of SARS-CoV-2 in child care facilities and to facilitate a timely public health response to prevent outbreaks.

**Summary**

**What is already known about this topic?**

COVID-19 cases reported at child care facilities are correlated with level of community transmission.

**What is added by this report?**

Among 469 child care facilities in the District of Columbia, 23.9% reported at least one COVID-19 case, and 5.8% reported outbreak-associated cases during July 1–December 31, 2020. Among 319 cases, approximately one half were among teachers or staff members. Outbreak risk was increased in facilities operating <3 years, with symptomatic persons who sought testing ≥3 days after symptom onset, or with asymptomatic cases.

**What are the implications for public health practice?**

Implementation and maintenance of multiple prevention strategies are important to reduce SARS-CoV-2 transmission in child care facilities and to facilitate a timely public health response to prevent outbreaks.

## Footnotes

* These authors contributed equally to this report.

† The Council of State and Territorial Epidemiologists defines an outbreak as two or more laboratory-confirmed COVID-19 cases among students or staff members with onset of illness within a 14-day period, that are epidemiologically linked, among persons who do not share a household, and are not listed as a close contact of each other in another setting during standard case investigation or contact tracing.

§ A COVID-19 case was defined as a positive nucleic acid amplification test, including positive reverse transcription–polymerase chain reaction test, or positive rapid antigen test result for SARS-CoV-2, in a person who was physically present at a child care facility. Symptomatic persons who did not have laboratory confirmation of infection were not included.

** CDC’s definition for cohorts was used (https://www.cdc.gov/coronavirus/2019-ncov/community/schools-childcare/schools.html). Examples of not adhering to cohorts are adding persons to cohorts that they are not assigned to or combining cohorts because of limited staffing.

†† Phase 2 guidance was updated in mid-December 2020.

§§ Floating teachers or staff members are those who leave their assigned cohort to cover for another teacher or staff member who might be absent or on break.

¶¶ Close contacts were defined as persons exposed to an index patient at a facility within 6 ft for ≥15 minutes during a 24-hour period while the index patient was infectious (48 hours before through 10 days after symptom onset or, if asymptomatic, 48 hours before through 10 days after specimen collection). DC Health’s approach was to quarantine the class or cohort to which the index patient belonged whenever the person was physically present at the facility during their infectious period, given the limited capacity to maintain physical distancing. Quarantine up to 14 days was recommended for all close contacts; follow-up was conducted by DC Health’s contact tracing team.

***
https://osse.dc.gov/publication/child-development-facilities-listing

††† Based on the date when a facility reported being notified of a positive SARS-CoV-2 test result and date when a consult form was submitted to DC Health.

§§§ 45 C.F.R. part 46.102(l)(2), 21 C.F.R. part 56; 42 U.S.C. Sect. 241(d); 5 U.S.C. Sect. 552a; 44 U.S.C. Sect. 3501 et seq.

¶¶¶ Asymptomatic persons with COVID-19 when interviewed by a case investigator or followed up with public health monitoring if they were DC residents.
